# Editorial: Methods in cellular neurobiology research

**DOI:** 10.3389/fncel.2025.1713920

**Published:** 2025-10-28

**Authors:** Sue Han, Adalberto Merighi, Cheng Wang, Shrijeet Chakraborti

**Affiliations:** 1 National Institutes of Neurological Disorders and Stroke, Bethesda, MD, United States; 2 Department of Veterinary Sciences, University of Turin, Grugliasco, TO, Italy; 3 Department of Neurodegenerative Diseases, City of Hope National Medical Center, Beckman Research Institute, Duarte, CA, United States; 4 Royal Preston Hospital, Preston, United Kingdom

**Keywords:** dorsal horn excitability, microcontact printing, organotypic slice culture, iPSC-derived neurons, microfluidic compartmentalization, extracellular vesicles, *C. elegans*, neurodegeneration

Cellular neuroscience has long relied on methodological innovation to reveal the nervous system's intricate structure and function. From Ramón y Cajal's use of Golgi staining method and the rise of electrophysiological recording to today's advanced imaging modalities and animal models, new tools have consistently propelled the field forward, enabling observations of the nervous system with ever-greater resolution and specificity. Landmark developments such as patch-clamp electrophysiology, two-photon microscopy, and genetically encoded calcium and voltage indicators have enabled detailed analyses of neuronal and glial physiology at unprecedented resolution (Denk et al., 1990; Chen et al., 2013; Luo et al., 2018). Complementary methods, including single-cell transcriptomics and advanced imaging technologies, now allow researchers to capture both the molecular and functional heterogeneity of neural cells. These approaches continue to drive forward our knowledge of excitability, connectivity, and plasticity under normal physiological conditions.

At the same time, methodological progress has transformed disease-focused neuroscience. Human induced pluripotent stem cell (iPSC)-derived neurons, motor neurons, and glia cells provide patient-specific models for studying pathogenic mechanisms and testing potential therapies (Takahashi and Yamanaka, 2006; Lancaster et al., 2013). Microfluidic devices and organotypic slice preparations enable controlled exploration of axonal degeneration, glial–vascular interactions, and long-term cellular dynamics. Similarly, model organisms such as *Caenorhabditis elegans* offer powerful systems for dissecting conserved mechanisms of neurodegeneration (Bargmann, 1998; Reilly et al., 2020). These methods have been critical in investigating diseases ranging from amyotrophic lateral sclerosis (ALS) and stroke to Alzheimer's disease (AD), Parkinson's disease (PD), and chronic pain syndromes. At the molecular level, methods for probing neurodegenerative-linked protein aggregates, including amyloid-β peptide, tau protein, and α-synuclein, have provided crucial insights into the cellular basis of AD, PD, and related disorders (Hardy and Selkoe, 2002; Spillantini and Goedert, 2013). Advances in glial biology, from astrocytic imaging to extracellular vesicle analysis, further demonstrate how methodological innovation is reshaping our understanding of non-neuronal contributions to disease.

This Research Topic, *Methods in Cellular neurobiology research*, brings together contributions that exemplify the breadth and creativity of current methodological approaches. Collectively, these studies illustrate how technical innovation not only expands our experimental repertoire but also drives conceptual advances across the spectrum of cellular neuroscience, from basic mechanisms of excitability to translational models of neurological disease. Why now? In neuroscience, there are three bottlenecks that hinder progress: (1) visualization of the right cell and structures with sufficient spatiotemporal resolution; (2) testing mechanisms in complementary model systems; and (3) turning mechanistic insights into better biomarkers, targets, or interventions of neurological disorders. The five articles in this Topic contribute to each bottleneck from a different angle.

The Research Topic begins with a comprehensive review, “*An electrophysiologist's guide to dorsal horn excitability and pain*.” Rivera-Arconada et al. synthesize intrinsic and synaptic determinants of dorsal horn firing (Na^+^, K^+^, HCN, GIRK, Ca^2+^-activated K^+^ currents), emphasizing strong cellular heterogeneity and the limits of predicting biochemical phenotype or morphology from firing patterns. This framework guides interpretation of circuit plasticity in injury and inflammation. In another method paper, Humpel introduces a microcontact-printing method that deposits antibodies for GFAP and laminin directly onto 150μm organotypic slices, enabling week-scale live imaging on standard inverted microscopes. The approach supports patterned, local delivery, lowers costs, and reduces animal use, expanding access to vascular–glial studies. In a research article, Otomo et al. use a compartmentalized microfluidic chip to examine early axonal phenotypes in iPSCderived motor neurons carrying FUS/TLS mutations. By DIV7 they detect growth restriction and altered mitochondrial trafficking; viability remains comparable early but declines by days 14 and 21, and stress worsens deficits. The platform yields tractable readouts for mechanism and drug discovery. In another interesting research article, Zhang et al. report that memantine-preconditioned MSC-derived extracellular vesicles (EVs), enriched in miR-139-5p and miR-133b, outperform conventional EVs in a mouse photothrombotic stroke model. These EVs improve functional recovery, reduce infarct burden, mitigate excitotoxicity, and activate neuroregenerative pathways, illustrating microenvironment-tailored EV engineering. Finally, Torres et al. review *C. elegans* as a fast, genetically precise system to interrogate proteinopathy, aging, and neuron–glia interactions. Tools such as transgenics, inducible expression, and pharmacology create efficient pipelines that bridge *in vitro* assays and mammalian studies. Together, these contributions span ionic mechanisms, accessible imaging platforms, disease modeling, and translational strategies, offering practical methods and conceptual clarity for probing and modulating neural systems in health and disease.

Taken together, this Research Topic shows how method innovation is already widening the three bottlenecks we outlined. Patterned microcontact printing is breaking the resolution barrier, making it possible to conduct weeks-long, low-cost live imaging of astroglia–vessel dynamics in organotypic slices. This gives standard microscopes routine access to glial biology and neurovascular remodeling. Utilizing a compartmentalized microfluidic platform, researchers can now perform precise mechanism testing by deriving quantitative axonal-phenotype assays from patient iPSC-derived motor neurons. This technique is powerful because it captures axonal growth and mitochondrial trafficking defects before degeneration, accelerating ALS research and drug screening. For translational impact, a targeted approach involves preconditioning mesenchymal-stromal-cell extracellular vesicles with an NMDAR inhibitor. This strategic modification reprograms the EV cargo for better efficacy, successfully helping to mitigate excitotoxicity and promote neurorepair in stroke models. It directly links mechanistic insight to therapeutic intervention. Model diversity is strengthened by a concise electrophysiological framework for dorsal horn excitability that anchors cellular heterogeneity to pain phenotypes, and by *C. elegans* pipelines that rapidly connect proteinopathy and neuron–glia interactions to conserved targets, streamlining paths from discovery to mammalian validation. Together, these methods equip cellular neuroscience to interrogate fundamental circuit rules, glia–vascular crosstalk, and neurodegenerative pathways, while building tractable routes from basic science to interventions across pain, stroke, and other neurological diseases. Overall, the work presented provides a strong foundation for future studies aiming to unravel the complexities of the nervous system in health and disease.
